# Supplementary material for: Chaperones rescue the energetic landscape of mutant CFTR at single molecule and in cell
Source: Nat Commun. 2017 Aug 30;8:398. doi: 10.1038/s41467-017-00444-4 (PMC5577305; doi:10.1038/s41467-017-00444-4)
Supplement: Supplementary file 1 — Supplementary Information [file 41467_2017_444_MOESM1_ESM.pdf]

File name: Supplementary Information
Description: Supplementary figures, supplementary tables and supplementary references.

File name: Peer review file
Description:

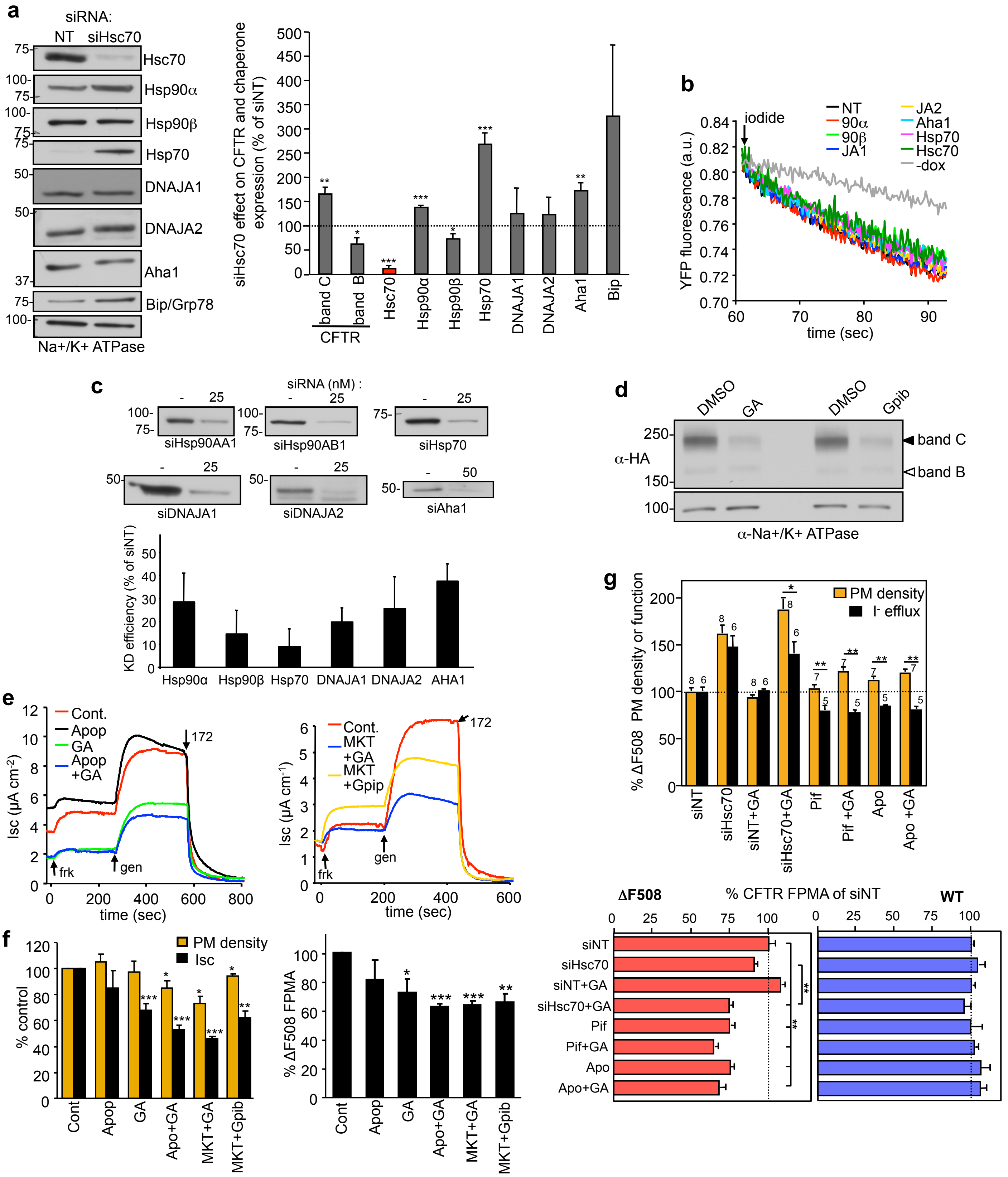

Supplementary Figure 1

Supplementary Figure 1. The role of molecular chaperones in the PM expression and function of ΔF508-CFTR in CFBE and HeLa cells.

(**A**) Hsc70 knock down (KD) effect on WT CFTR and chaperones expression in CFBE. Expression of CFTR and chaperones was detected by immunoblotting of cells transfected by 50 nM siHsc70 or NT siRNA (non-targeted). *Right panel:* Densitometric analysis of CFTR and chaperone/co-chaperone expression following Hsc70 KD in CFBE cells. Results show means ± SEM, n=2-3. $^{*}P<0.05$, $^{**}P<0.01$, $^{***}P<0.001$. (**B**) The effect of chaperone or co-chaperone KD on ΔF508-CFTR function in CFBE. Hsp90α (90α), Hsp90β (90β), DNAJA1 (JA1), DNAJA2 (JA2), Aha1, Hsp70, or Hsc70 were silenced with 50nM siRNA. Cells transfected with NT siRNA served as controls. Rescued ΔF508-CFTR (30°C, 48h) was unfolded (2h, 37°C) and its function was determined by halide-sensitive YFP quenching assay as described in Methods. (**C**) CFBE was transfected with specific or NT siRNA (-) and expression of each target gene was monitored by quantitative Western blotting on equal amounts of cell lysates (means ± SED, n=2-3, *lower panel*). **(D)** Inhibition of Hsp90 by Geldanamycin (5μM) or Genetispib (100nM) downregulates the complex- and core-glycosylated WT-CFTR expression (band C and band B, respectively) in CFBE after 24 hours. Na^+/K^+-ATPase was used as a loading control. (**E-F**) The effect of Hsc70 and Hsp90 acute inhibition on the PM density, function and FPMA of ΔF508-CFTR-3HA in filter grown CFBE. After rescuing ΔF508-CFTR (30°C, 48h), cells were exposed to 5 μM apoptozole (Apop), 5μg ml^{-1} geldanamycin (GA), 10 μM MKT-077 (MKT) or 100nM ganetespib (Gpib) individually or in combinations for 2h. Short circuit current (Isc) was measured after CFTR activation with forskolin (frk, 20 μM) and genistein (gen, 100 μM) followed by CFTR inhibition with Inh172 (172, 20 μM). Results show means ± SEM, n=4. $^{*}P<0.05$, $^{**}P<0.01$, $^{***}P<0.001$. (**G**) The effect of Hsc70 KD and chaperone inhibition on the PM density and PKA-activated chloride transport of ΔF508- and WT-CFTR-3HA, was determined by PM ELISA and iodide efflux, respectively, in HeLa cells. Hsc70

was KD with 50nM siHsc70. Rescued ΔF508-CFTR (26°C, 48h) was unfolded (2.5h, 37°C) in the absence or presence of Hsc70 (1 μM pifthrin μ [Pif] or 1 μM apoptozole [Apo]) or Hsp90 (10 μg ml^{-1} geldanamycin [GA]) inhibitor. PM density and function was expressed as percentage of NTsiRNA cells. Lower panels depict the FPMA of CFTRs. Results show means ± SEM, n=5-8. $^{*}P<0.05$, $^{**}P<0.01$, $^{***}P<0.001$.

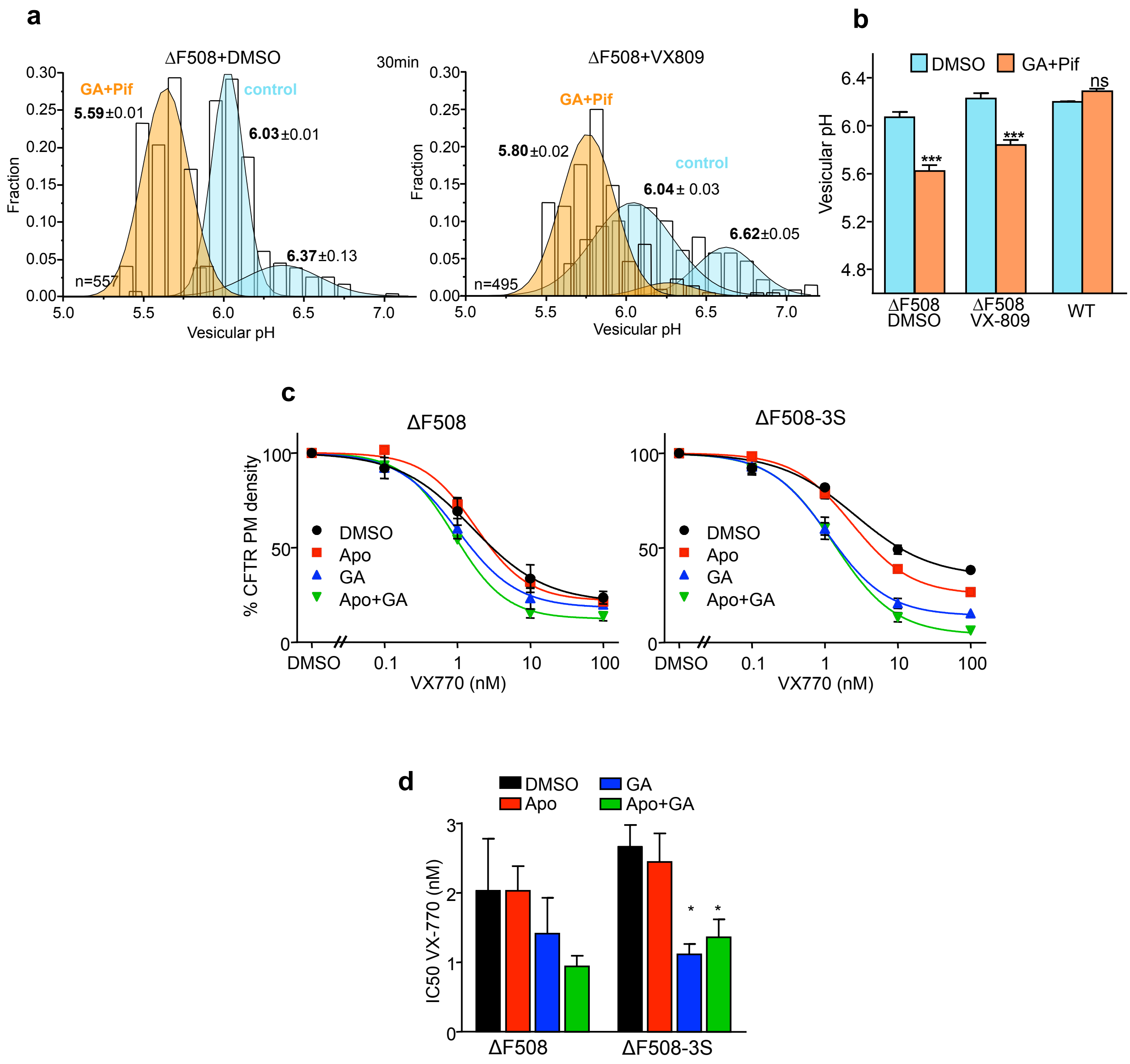

Supplementary Figure 2

Supplementary Figure 2. Modulation of ΔF508-CFTR biochemical stability at the PM and in post-endocytic compartments.

(A) Representative histograms of the luminal pH (pH_v) of ΔF508-CFTR-3HA containing endocytic vesicles. pH_v was measured by fluorescence ratiometric image analysis (FRIA), as described in Methods. GA and Pif (5μM each) were present during Ab incubation (0°C) and then the 30 min chase at 37°C. Cells were treated with DMSO (left panel) or VX-809 (3 μM, right panel) for 24h and during the chase. The pH_v distribution of ~500 endocytic vesicles is shown from one representative set of experiments of three. **(B)** The influence of GA+Pif on the endo-lysososomal distribution of internalized ΔF508-CFTR in control and VX-809 treated CFBE. Data are means ±SEM, n=3. (**C**) Chaperone inhibition sensitizes ΔF508- and ΔF508-CFTR-3S to downregulation by the gating potentiator, VX-770, after 24 h treatment in CFBE. Rescued ΔF508-CFTR (26°C, 48h) was unfolded for 30 min at 37°C prior to a 2h incubation with chaperone inhibitors (10 μM Apo, 10μM GA and combination). ΔF508-CFTR-3S cells were not rescued. CFTR PM density is expressed as percent of the control before VX-770 treatment. Data show means ±SEM, n=3. (**D**) The effect of Hsc70 and Hsp90 inhibition on the IC50 of VX-770-induced ΔF508-CFTR variant downregulation at the PM in CFBE. Experiments were performed as in panel C, using Apo+GA inhibitors as indicated. Data show means ±SEM, n=4.

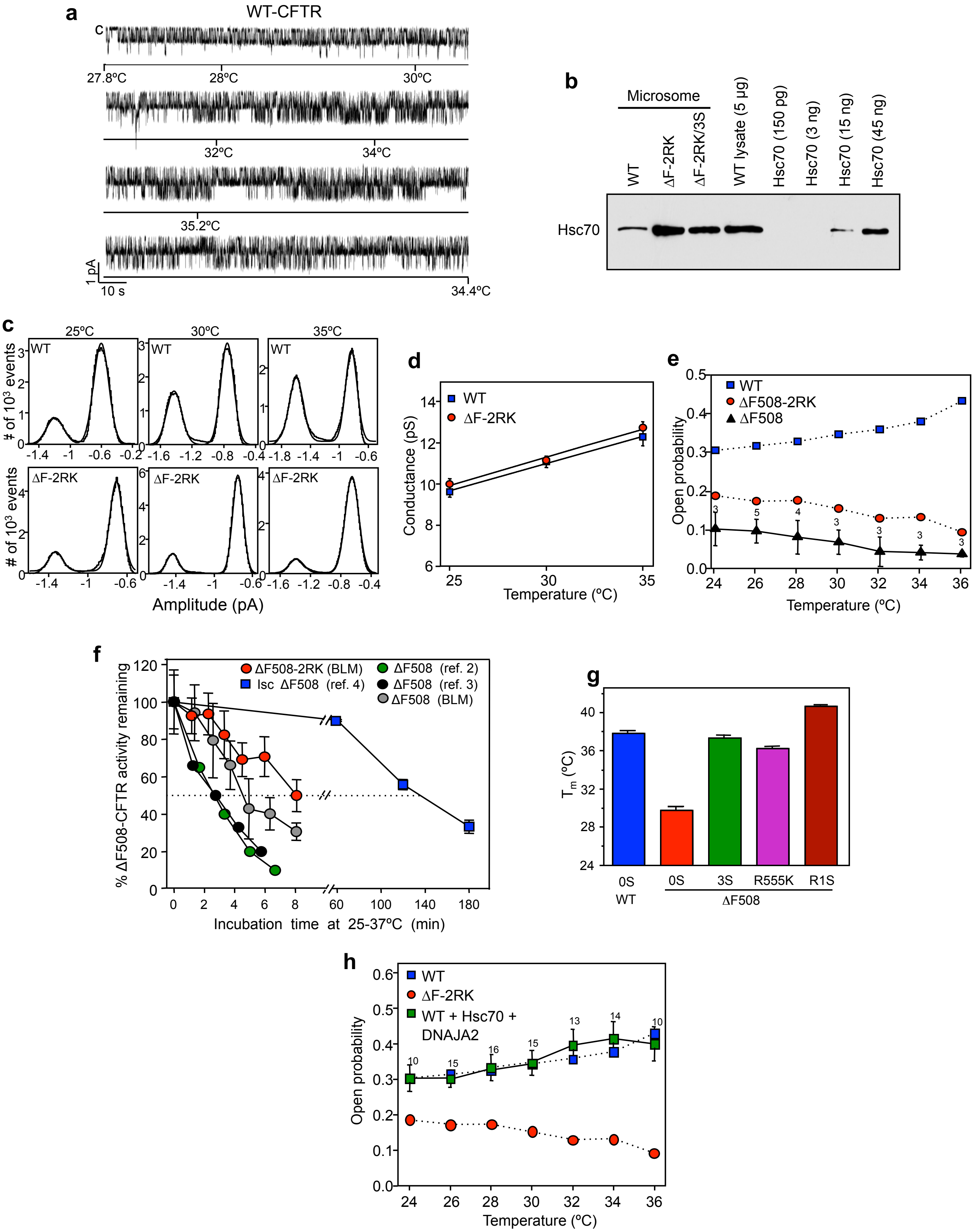

Supplementary Figure 3

Supplementary Figure 3. Functional and biochemical characterization of CFTR and NBD1 variants.

(A) WT-CFTR gating activity is preserved after a temperature ramp in the BLM. The activity of two WT-CFTR channels was monitored for ~6 min at ~34-35°C after the ramp. **(B)** The Hsc70 concentration in the BLM was negligible. The amount of Hsc70 that was co-fractionated with microsomes (~5μg), isolated from BHK-21 cells, expressing WT, ΔF508-CFTR-2RK, or ΔF508-2RK/3S-CFTR, was determined by immunoblotting. Purified Hsc70 (0.15-45 ng) and whole cell lysates were loaded as reference. The Hsc70 concentration in the BLM cup was ~40 nM, <1% of the cytoplasmic Hsc70 concentration (~5 μM)[1] (n=2). **(C)** All point amplitude histograms of WT and ΔF508-CFTR-2RK currents at 25, 30, and 35ºC from 1 min representative records in BLM. **(D)** Temperature-dependence of WT- and rΔF508-CFTR-2RK unitary conductance (pS) was calculated from the amplitude histogram ($g=i\,V_h^{-1}$, where i is the single channel amplitude and V_h is the holding membrane potential -60 mV, n=3-4). **(E)** The Po of WT-, rΔF508-, and rΔF508-CFTR-2RK was calculated during temperature ramps (~1.4°C min^{-1}) in the BLM. The Po values of the WT- and rΔF508-CFTR-2RK are shown (dashed lines) from Fig.3F. **(F)** Comparison of functional inactivation kinetics of rΔF508-CFTR at the PM, in excised patch and in the BLM. The ΔF508-CFTR inactivation kinetics in excised patch configuration was reported by the Kirk[2] and Sheppard[3] labs. The functional PM turnover of rΔF508-CFTR in CFBE cells was monitored by Isc measurements and derived from our publication[4]. Incubation time at 37°C is indicated, except for the BLM studies, where temperature ramps were imposed as in panel E. n values are as in Fig.3F, Supplementary Fig.3F-G and Isc n=3. **(G)** Thermal stability of isolated NBD1 variants, containing the indicated second site suppressor mutations (see Supplementary Table 1), was monitored by differential scanning fluorimetry (DSF) using SYPRO Orange and is depicted as melting

temperatures (T_m) (means ±SEM, n=3). **(H)** Hsc70 and DNAJA2 have no discernible effect on the WT-CFTR Po. Channel activity was recorded in the BLM. Chaperones were added at 2 μM concentration. Data are means ± SEM, n=10-16.

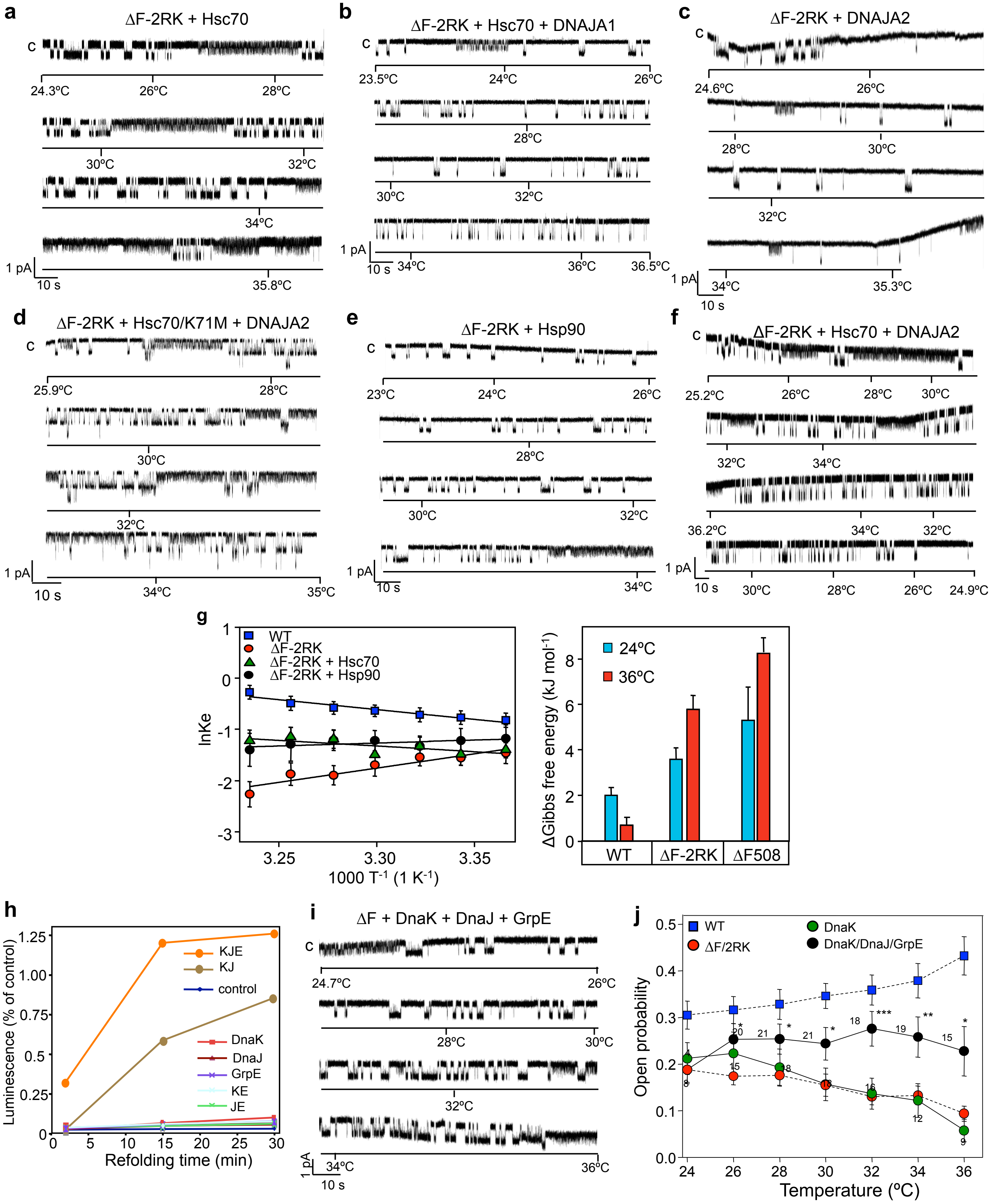

Supplementary Figure 4

Supplementary Figure 4. Co-chaperones are required for chaperone-mediated refolding of ΔF508-CFTR-2RK.

(A-E) Representative temperature-dependent gating activity of ΔF508-CFTR-2RK in BLM in the presence of Hsc70 (A), Hsc70 + DNAJA1 (B), DNAJA2 (C), Hsc70-K71M + DNAJA2 (D) and Hsp90β (E) as described in Fig.3. **(F)** Thermal inactivation of the ΔF508-CFTR-2RK is reversible. After the temperature ramp, the BLM temperature was reduced to 24ºC, which was coincided with regained activity of the mutant. Record only shows the channel gating during the second temperature ramp. The Po values were 0.13 and 0.16 at 25-30°C before and after the second temperature ramp, respectively. Representative record of 14 observations. **(G)** *Left panel:* Semi-logarithmic Van't Hoff plots of WT and ΔF508-CFTR-2RK gating in the presence of Hsc70/DNAJA2 or Hsp90/Aha1. Equilibrium gating constants were calculated from Po of single and multiple channel activities (n values are the same as in Fig.2F). Data were fitted by linear regression analysis. *Right panel*: Free energy of channel opening at 24ºC and 36ºC was calculated based on the $\Delta G_{O\text{-}C} = -RT\ln K_e$ equation as described in Methods. (**H)** Folding activity of purified DnaK, DnaJ, and GrpE . Denatured firefly luciferase (8μM) in 6M Gdm-Cl (5 min, 26ºC) was after 100-fold dilution into 40 mM Hepes/KOH pH 7.5, 50mM K-acetate, 2 mM Mg-acetate$_2$, and 2 mM ATP at 30°C in the absence or presence of DnaK (K, 800nM), DnaJ (J, 160nM) and GrpE (E, 400nM). Luciferase activity was measured by luminometry in the presence of 70μM D-luciferin and 5 mM ATP and expressed as the percentage of non-denatured luciferase activity (n=3). (**I**) Temperature-dependent single-channel activity of rΔF508-CFTR-2RK in the presence of DnaK (1 μM), DnaJ (0.2 μM) and GrpE (0.5 μM). (**J**) The effect of DnaK or DnaK/DnaJ/GrpE on the Po of ΔF508-CFTR-2RK, determined as the function of temperature. Results show means ± SEM. $^{*}P<0.05$, $^{**}P<0.01$, $^{***}P<0.001$.

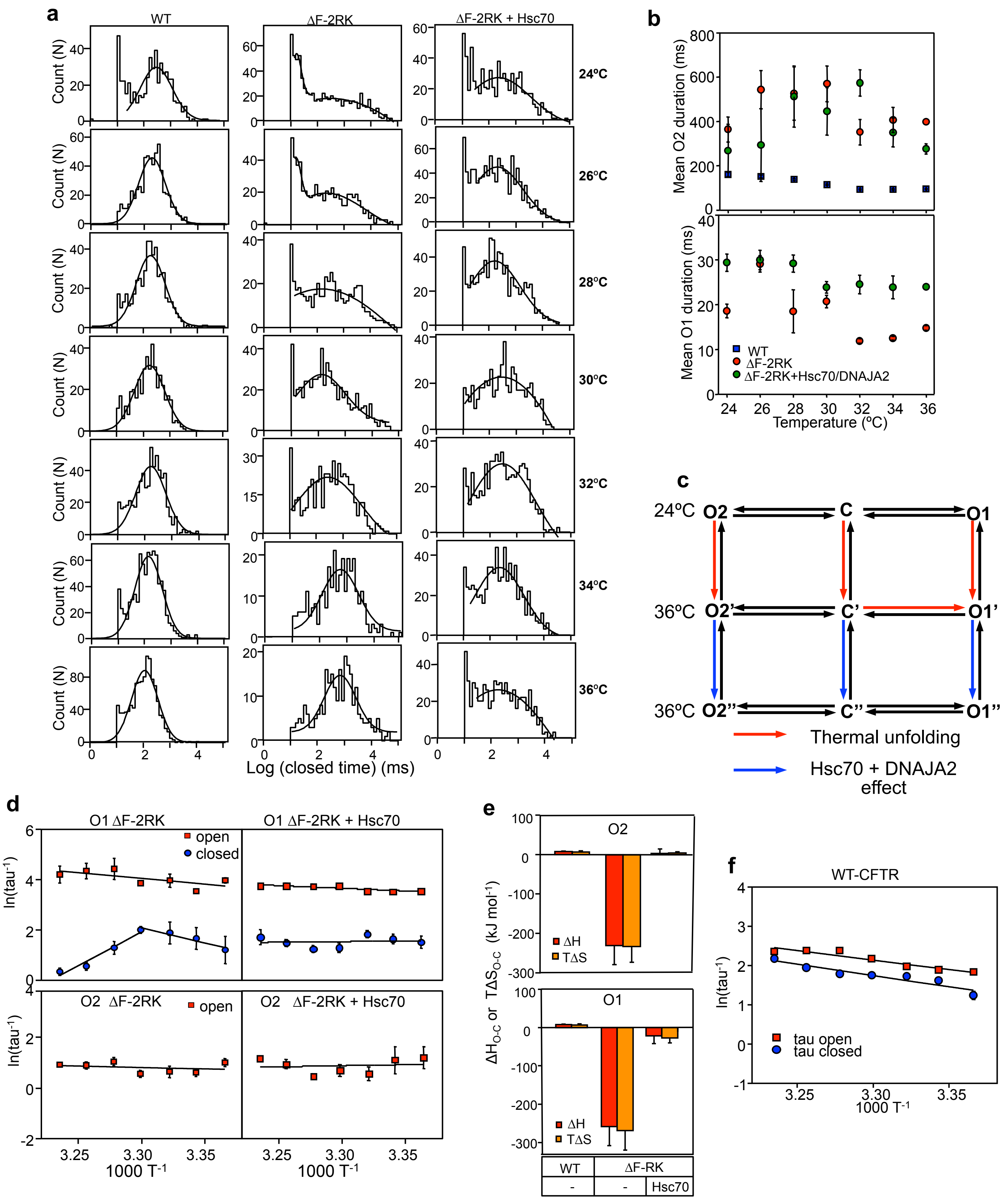

Supplementary Figure 5

Supplementary Figure 5. Effect of molecular chaperones on the temperature-dependent gating kinetics and energetics of WT- and ΔF508-CFTR.

(A) The temperature effect on closed time histograms of WT- and ΔF508-CFTR-2RK with or without Hsc70/DNAJA2. Peaks were fitted with one Gaussian distribution to the following single channel records: ΔF508-CFTR-2RK (total duration=10-28 min, n=5-24), ΔF508-CFTR-2RK with Hsc70/DNAJA2 (15-25 min, n=10-23) and WT (4-7 min, n=3-7), except for the ΔF508-CFTR-2RK at 24 and 26ºC, where two Gaussian distributions were fitted. **(B)** Temperature-dependence of the mean O1 and O2 time of WT, ΔF508-CFTR-2RK and ΔF508-CFTR-2RK in the presence of Hsc70/DNAJA2 based on dwell time histograms obtained from records as in panel A **(C)** A simplified gating model of temperature rescued ΔF508-CFTR-2RK consists of three states: $O1_{(T)}$ - $C_{(T)}$ - $O2_{(T)}$ at 24°C. Upon thermal unfolding these states are transformed into O1', O2' and C' states in the BLM at 36°C. Unfolding is characterized by the increased prevalence of short O1' and longer C states (see Fig. 4, Fig. S4 and Table S2). During thermal unfolding, the partially unfolded channel is recognized by the Hsc70/DNAJA2 complex, which remodels the polypeptide folding trajectory by entropic pulling and engaging the channel in unfolding/refolding cycle. This leads to the shift of the O1', O2' and C' states toward near-native, temperature-rescued ΔF508-CFTR-2RK conformations (O1'', O2'' and C'') (blue arrows), reflected by the partially restored channel gating (Fig. 2F). While the gating kinetics analysis revealed energetic differences between the open (O1 and O2) states, the conformational and energetic differences between C, C' and C'' states cannot be defined. Considering that chaperone inhibition has an effect on ΔF508-CFTR FPMA in the absence of PKA activation (see Fig.2C), we propose that both the open and the closed states are subjected to conformational rearrangement by chaperones (blue arrows). This is arbitrarily indicated by the increased free energy of the ΔF508-CFTR-2RK closed state upon thermal unfolding at 36°C and its reduction by Hsc70/DNAJA2 activity (see Fig. 5B), which suggest that the chaperone-induced energetic stabilization of the ΔF508-CFTR-2RK is probably underestimated at 36°C. **(D)** Arrhenius

plots of short (O1, top panels) and long (O2, lower panels) opening, as well as closing rate constants calculated from single channel gating activity. Data were fitted by linear regression. (**E**) Steady-state enthalpic and entropic energy changes required for the ΔF508-CFTR C to O1 or O2 transitions and the effect of Hsc70/DNAJA2 at 36°C, calculated based on the Arrhenius plots of panels D and F. WT CFTR was used as reference. **(F)** Arrhenius plots of WT CFTR opening and closing transition rates as a function of temperature. Data were fitted by linear regression. Rate constants were determined from mean open and closed times as described in Methods. Results are means ± SEM, n=5-24 (ΔF), n=10-23 (ΔF with Hsc70/DNAJA2) and n=3-7 (WT).

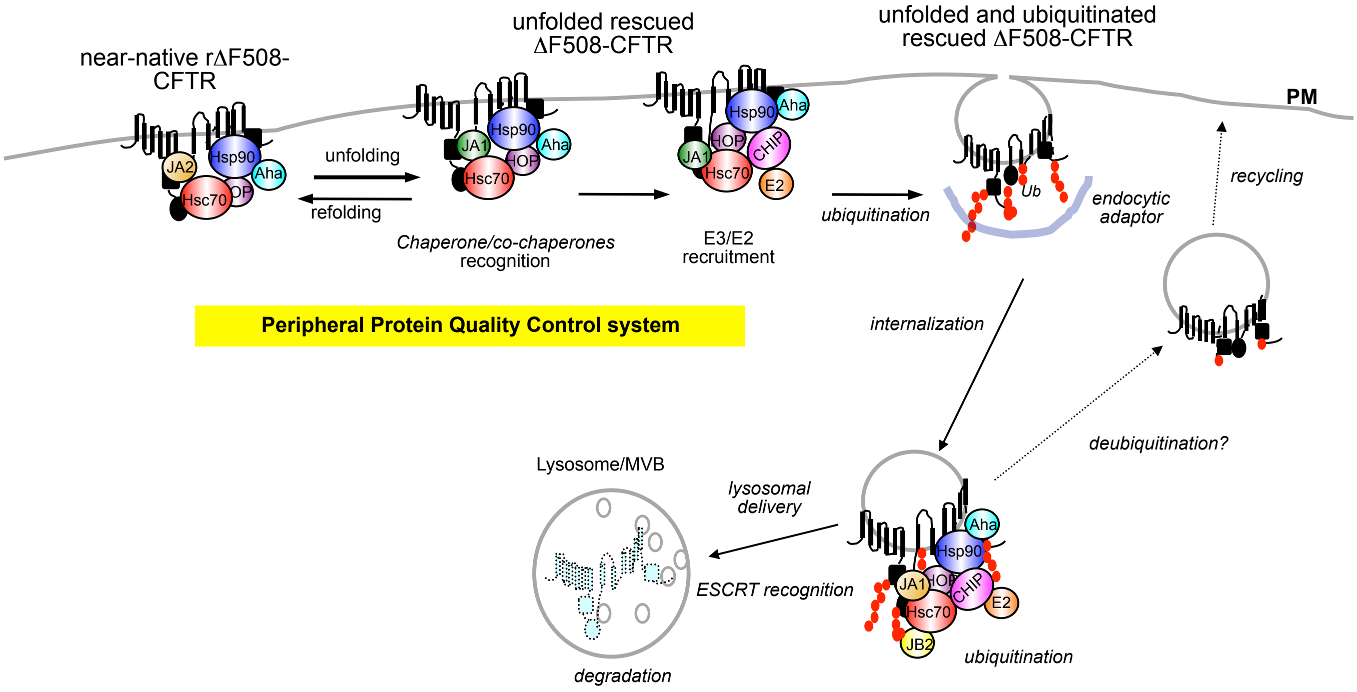

Supplementary Figure 6

Supplementary Figure 6. Schematic model of molecular chaperones role in refolding and degradation of near-native CFTR at the PM.

Molecular chaperone and co-chaperone complexes can contribute to both the conformational maintenance and degradation of ΔF508-CFTR at the PM. Depending on the severity of the channel conformational defect and the cellular proteostasis network activity and composition, the pro-folding or the pro-degradative function of associated chaperone complexes will be dominant at the PM and endocytic compartments. Although both Hsc70 and Hsp90 contribute to the refolding and ubiquitination of the unfolded ΔF508-CFTR, DNAJA2 is preferentially required for the mutant refolding and DNAJA1 is necessary for its ubiquitin-dependent lysosomal downregulation[5] from the PM, suggesting distinct co-chaperone requirement for the pro-degradative and pro-folding roles of chaperones.

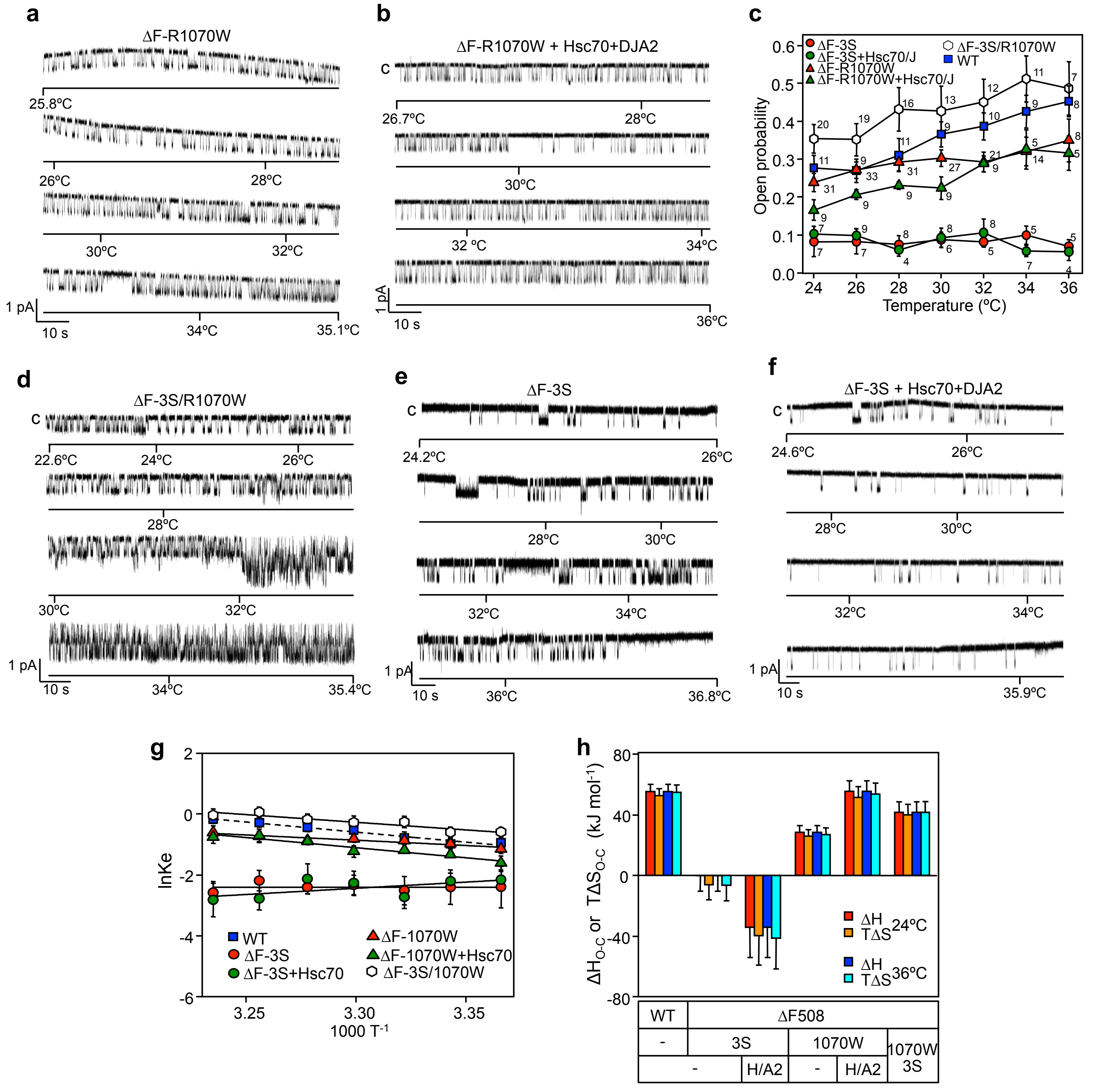

Supplementary Figure 7

Supplementary Figure 7. The impact of second-site suppressor mutations on ΔF508-CFTR-3HA susceptibility to Hsc70 meditated folding.

The influence of NBD1 and/or NBD1-MSD2 second site suppressor mutations (3S and R1070W, respectively) on the temperature-dependent ΔF508-CFTR gating was measured in BLM. **(A-F)** Representative channel activity of ΔF508-CFTR-R1070W in the absence (A) and presence (B) of Hsc70/DNAJA2, as well as ΔF508-CFTR-3S/R1070W (D) and ΔF508-CFTR-3S in the absence (E) and presence (F) of Hsc70/DNAJA2, are shown. (C) Open probabilities of ΔF508-CFTR, containing the indicated stabilizing mutations in the presence or absence of Hsc70/DNAJA2 (Hsc70/J). **(G-H)** Energetic stabilization of ΔF508-CFTR open state by NBD1-MSD2 interface (R1070W), but not the NBD1 suppressor 3S mutation. Semi-logarithmic Van't Hoff plots depicts the temperature-dependence of the gating equilibrium constant $\ln(K_e)$, calculated from the Po for the mutants. Data were fitted by linear regression and plots were used to calculate steady-state energetic parameters (G). $\Delta H_{O\text{-}C}$ and $T\Delta S_{O\text{-}C}$ values at 24°C and 36°C were determined from the slope and intercept, respectively, as described in the Methods (H). Data are means ± SEM, n values are the same as in Figure 7A.

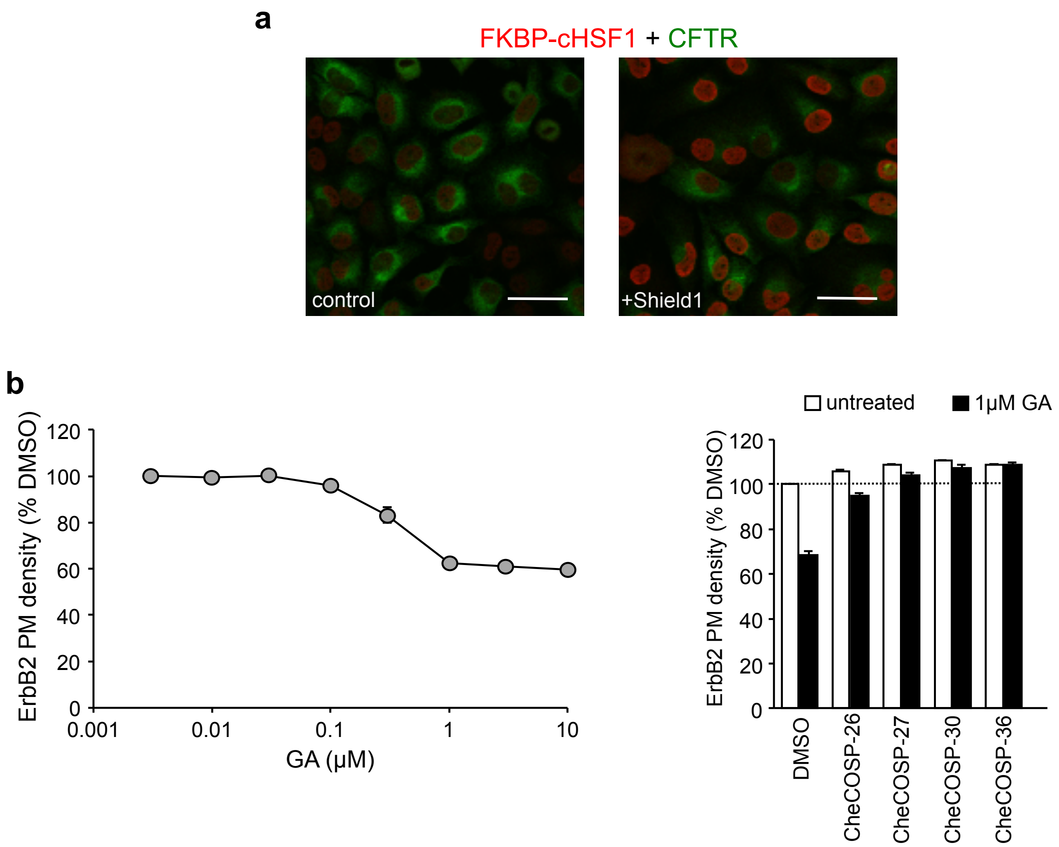

Supplementary Figure 8

Supplementary Figure 8. Modulation of HSF1 expression and the effect of Hsp90 allosteric activators on the ErbB2 PM turnover.

(A) Co-expression of FKBP-cHSF1 with ΔF508-CFTR-3HA in HeLa cells was visualized by indirect immunostaining with anti-HSF1 and anti-HA primary antibodies, respectively, before and after stabilization of FKBP-cHSF1 by Shield1. Bar: 50 μm. (**B**) Geldanamycin (GA) downregulated ErbB2 from the PM for CFBE (left panel), monitored by cell surface ELISA. PM ErbB2 was labelled by anti-ErbB2 Ab on ice. After 1.5 h incubation at 37°C in the presence of GA, the remaining ErbB2 receptor PM density was determined. Right panel: Hsp90 activators can counteract the GA-induced downregulation of the ErbB2 receptor from the PM, measured by cell surface ELISA. The receptor turnover was measured in the absence or presence of the indicated Hsp90 allosteric activators (50 μM) after 1.5 h incubation at 37°C with 1 μM GA. Data are means ± SEM, n=3.

Fig. 1A (Hsc70)

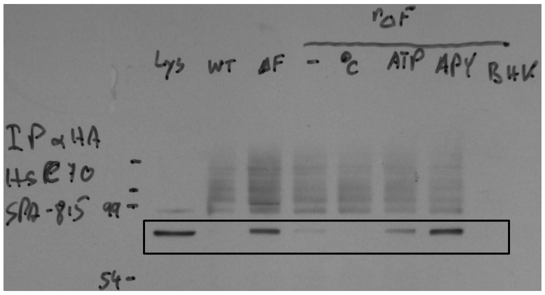

Fig. 1A (Hsp90)

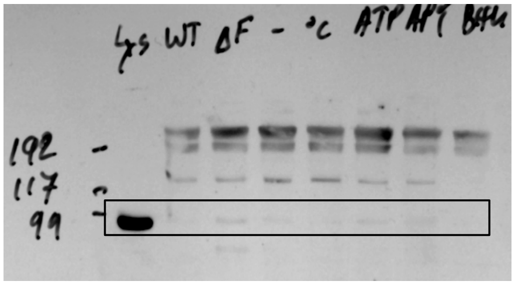

Fig. 1A (HA)

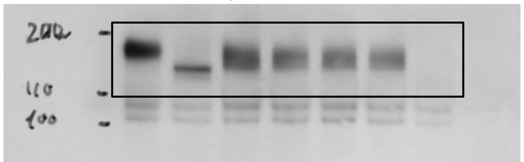

Fig. 1C (Aha1 and Hdj1)

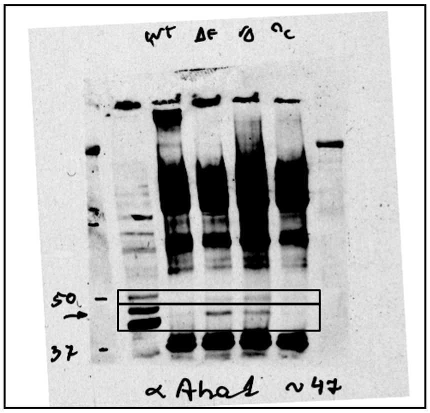

Fig.1D

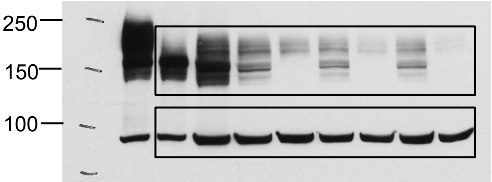

Fig.1E DMSO control

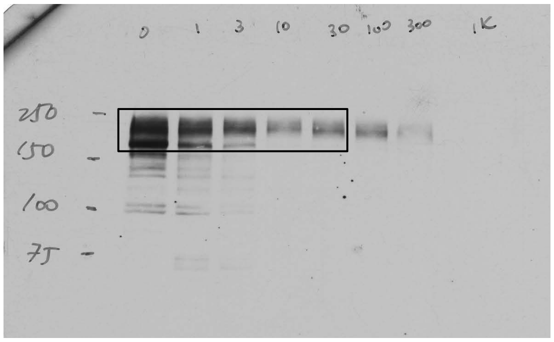

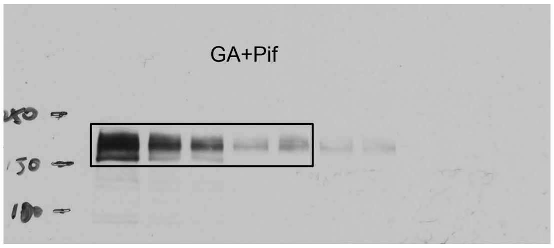

Supplementary Figure 9

Fig. 8A

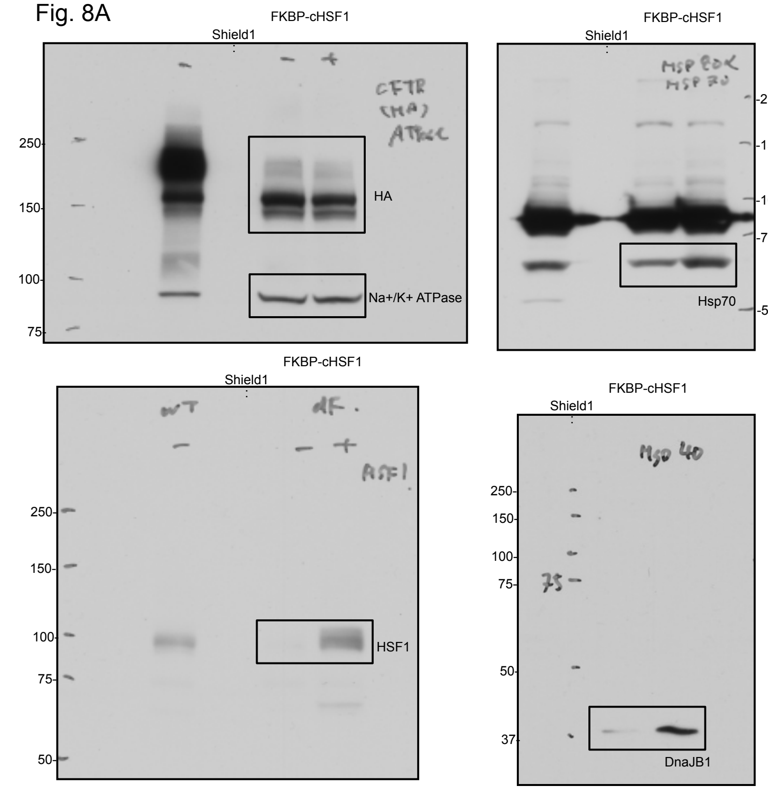

Fig. S1A

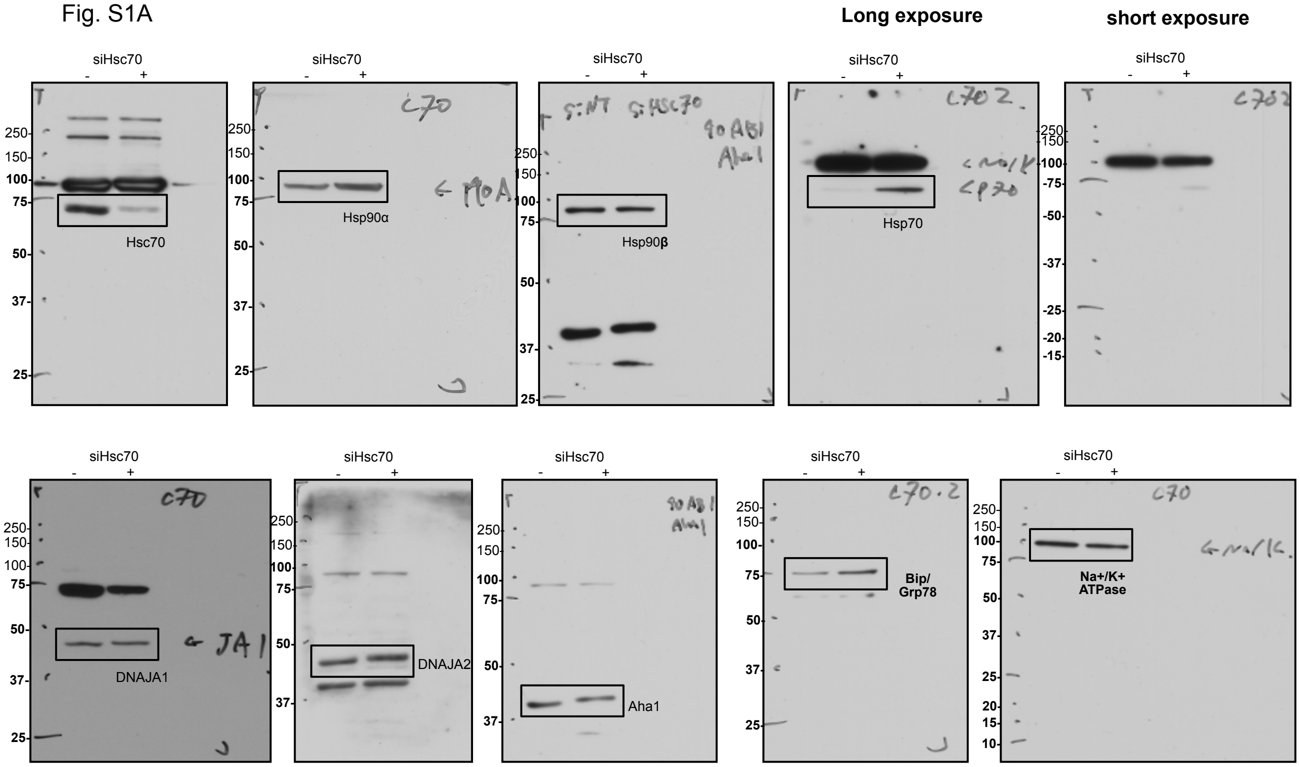

Supplementary Figure 9 cont’d

Fig. S1C

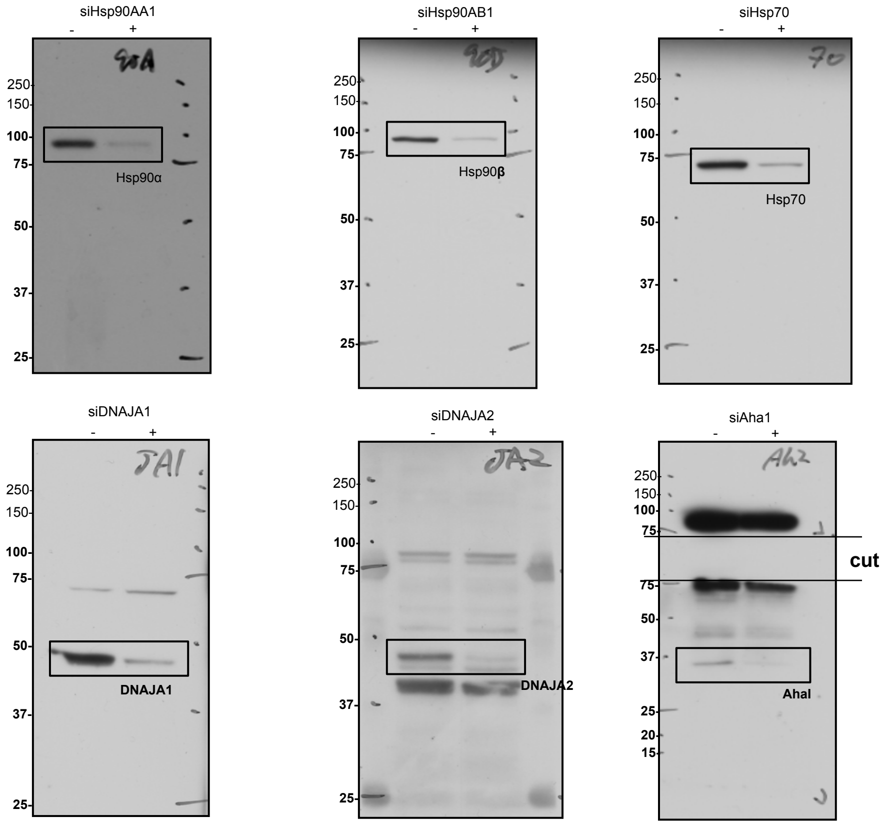

Fig. S3B

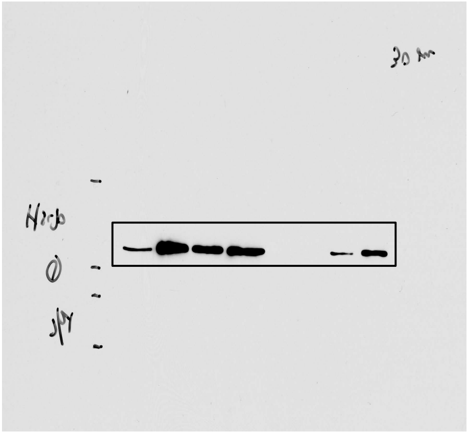

Fig. S1D

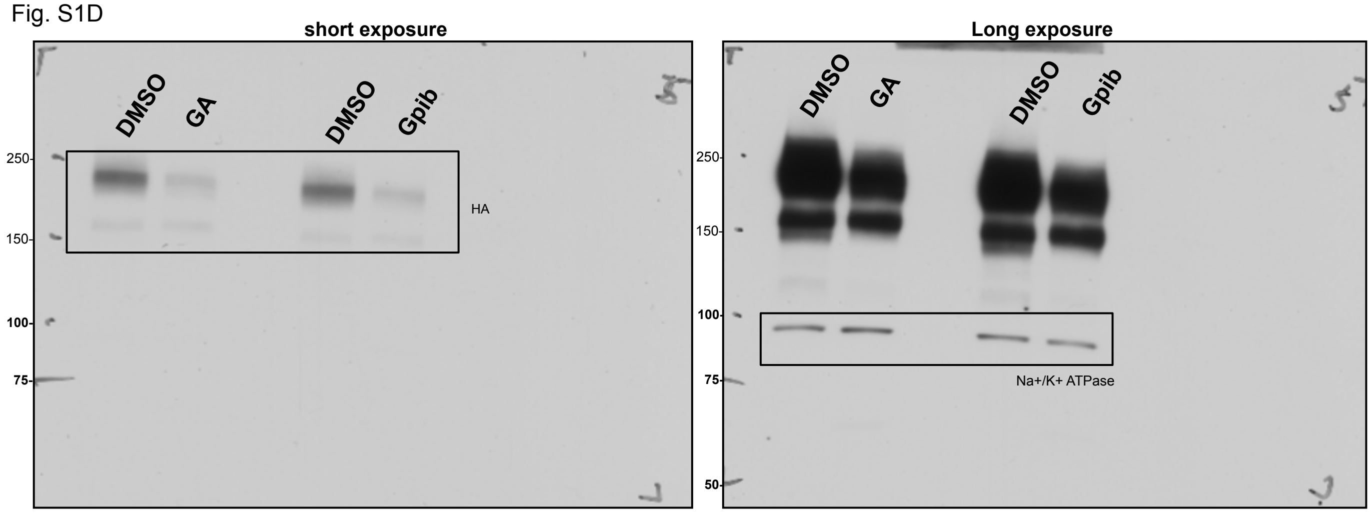

Supplementary Figure 9 cont'd

Supplementary Figure 9. Uncropped scans of the western blots.

Full size scans of western blots accompanied by size markers. Black boxes show the region presented in the indicated figures.

SUPPLEMENTARY TABLES

Supplementary Table 1. Variants of CFTR and NBD1 used

CFTR variants	Second site mutation	Tag	Target#	Ref.
WT CFTR		3HA		
WT CFTR		CiHA		
ΔF508		3HA		
ΔF508		CiHA		
ΔF508-R29K	R29K	3HA	ER retention	[6, 7]
ΔF508-R555K	R555K	3HA	NBD1, NBD1-NBD2	[6, 7]
ΔF508-R555K	R555K	CiHA	see above	[6, 7]
ΔF508-2RK	R29K, R555K	3HA	see above	[6, 7]
ΔF508-2RK	R29K, R555K	CiHA	see above	[6, 7]
ΔF508-3S	F494N, Q637R, F429S	3HA	NBD1	[8, 9]
ΔF508-2RK-3S	R29K, R555K, F429S, F494N, 637R	CiHA	NBD1, NBD1-NBD2	[6, 7, 8]
ΔF508-R1070W	ΔF508	3HA	NBD1-MSD2	[9, 10]
ΔF508-3S-R1070W	F429S, F494N, Q637R, R1070W	3HA	see above	[8, 9, 11, 12]
ΔF508-R1S	ΔF508, G550E, R553Q, R555K, F494N	3HA	NBD1, NBD1-NBD2	[4, 6, 7, 8, 9, 10, 13, 14]
ΔF508-E1371S	E1371S	3HA	NBD1-NBD2	[4, 15, 16, 17]
P67L		3HA		[18]

NBD1 variants	second-site mutation	Effect	Ref.
WT 0S	none		
ΔF508-0S	none		
ΔF508-3S	F429S, F494N, Q637R	NBD1 stab.	[9, 19]
ΔF508-R555K	R555K	see above	[9, 19]
ΔF508-R1S	G550E, R553Q, R555K, F494N	NBD1 and NBD1-NBD2 stab.	[9, 19]

#Target: the indicated second site mutation probably stabilizes the following structure: ΔF508-NBD1, ΔF508-NBD1-MSD2 and/or ΔF508-NBD1-NBD2 interface.

Supplementary Table 2. Temperature dependence of mean open and closed times and gating rate constants

T (° C)		tau O1 (ms)	%	tau O2 (ms)	%	ko_1-c	SEM	ko_2-c	SEM
24	WT			160 ± 9.2				6.2	0.4
	ΔF/2RK	18.6 ± 1.5	42.7	364 ± 56.6	57.3	52.6	5.5	2.7	0.4
	ΔF/2RK+Hsc70	29.4 ± 1.9	38.9	268.1 ± 118.2	61.1	34	2.3	3.7	1.6
26	WT			151.3 ± 5.8				6.6	0.3
	ΔF/2RK	29.1 ± 1.8	66.9	542.9 ± 86.2	33.1	34.5	2.4	1.8	0.3
	ΔF/2RK+Hsc70	30 ± 2.2	52.1	293.5 ± 164.2	47.9	33.4	2.4	3.4	1.9
28	WT			138.1 ± 4.1				7.2	0.2
	ΔF/2RK	18.5 ± 4.8	83	525.7 ± 120.7	17	52.6	13.9	1.9	0.4
	ΔF/2RK+Hsc70	29.2± 1.9	58.4	512.3 ± 138.1	41.6	34.3	2.3	2	0.5
30	WT			114.2± 3.3				8.8	0.3
	ΔF/2RK	20.7 ± 1.4	58	569.7 ± 81.1	42	47.6	2.3	1.8	0.2
	ΔF/2RK+Hsc70	23.9 ± 1.3	57	445.4 ± 107.7	43	41.9	2.3	2.2	0.5
32	WT			93.2 ± 2.1				10.7	0.2
	ΔF/2RK	11.9 ± 0.5	63.4	351.8 ± 57.5	36.6	83.3	34.7	2.8	0.5
	ΔF/2RK+Hsc70	24.5 ± 2.1	55.4	573.5 ± 59.3	44.6	40.8	3.4	1.7	0.2
34	WT			93 ± 2.8				10.8	0.3
	ΔF/2RK	12.5 ± 0.4	65.6	406.7 ± 56.8	31.4	76.9	23.7	2.5	0.3
	ΔF/2RK+Hsc70	23.9 ± 2.5	43.6	349.9 ± 64.9	56.4	41.9	4.5	2.9	0.5
36	WT			94.8 ± 2.4				10.5	0.3
	ΔF/2RK	14.8 ± 0.5	67.2	397.8 ± 0	32.8	66.7	22.2	2.5	0
	ΔF/2RK+Hsc70	24 ± 0	23.7	276 ± 23	76.3	41.7	0	3.6	0.3

T (° C)		tau C1 (ms)	%	tau C2 (ms)	%	kc_1-o	SEM	kc_2-o	SEM
24	WT			289.1 ± 30.4	100			3.5	0.4
	ΔF/2RK	17.1 ± 0.5	41.7	296.2 ± 159.9	58.3	58.8	3.5	3.4	1.8
	ΔF/2RK+Hsc70			216.8 ± 52.1	100			4.6	1.1
26	WT			197.6 ± 10.2	100			5.1	0.3
	ΔF/2RK	15.7 ± 1	32.6	206.6 ± 84.9	67.4	66.7	4.4	5.3	2.3
	ΔF/2RK+Hsc70			191.7 ± 33.9	100			5.2	0.9
28	WT			178.3 ± 10.6	100			5.6	0.3
	ΔF/2RK			150.5 ± 64.4	100			6.6	2.8
	ΔF/2RK+Hsc70			159 ± 23.5	100			6.3	0.9
30	WT			173.4 ± 11	100			5.8	0.4
	ΔF/2RK			134.3 ± 18.4	100			7.4	1
	ΔF/2RK+Hsc70			269.2 ± 52.3	100			3.7	0.7
32	WT			166.85 ± 11.8	100			6	0.4
	ΔF/2RK			271.4 ± 69.2	100			3.7	0.9
	ΔF/2RK+Hsc70			284.2 ± 39.7	100			3.5	0.5
34	WT			142.5 ± 8.1	100			7	0.4
	ΔF/2RK			562.7 ± 88.9	100			1.8	0.3
	ΔF/2RK+Hsc70			223.1 ± 30.4	100			4.5	0.6
36	WT			114 ± 5.4	100			8.8	0.4
	ΔF/2RK			700.6 ± 104.6	100			1.4	0.2
	ΔF/2RK+Hsc70			179 ± 53	100			5.6	1.7

Tau O1 and O2 are open dwell times, tau C1 and C2 are closed dwell times of the WT- and ΔF508-CFTR-2RK. $k_{O1\text{-}C}$ and $k_{O2\text{-}C}$ are transitional rate constants of closing, while $k_{C1\text{-}O}$ and $k_{C2\text{-}O}$ are transitional rate constants of opening.

Supplementary Table 3. Gating transitions between O1 and O2 as a function of temperature

T (° C)		# O1-O2	% O1-O2	# O2-O1	% O2-O1	total event
24	ΔF/2RK	3	0.4	2	0.3	1444
	ΔF/2RK+Hsc70	8	1.1	4	0.5	1462
26	ΔF/2RK	8	1.3	2	0.3	1248
	ΔF/2RK+Hsc70	8	0.8	6	0.6	2121
28	ΔF/2RK	8	1.6	6	1.2	974
	ΔF/2RK+Hsc70	12	1.5	6	0.7	1649
30	ΔF/2RK	8	1.3	3	0.5	1248
	ΔF/2RK+Hsc70	4	0.7	5	0.9	1104
32	ΔF/2RK	3	0.6	3	0.6	1018
	ΔF/2RK+Hsc70	7	1.2	5	0.9	1159
34	ΔF/2RK	4	1.3	3	1	606
	ΔF/2RK+Hsc70	12	1.7	10	1.4	1446
36	ΔF/2RK	1	0.4	1	0.4	526
	ΔF/2RK+Hsc70	3	0.5	8	1.2	1344

O1-O2 and # O2-O1 indicate the number of single channel gating transitions from the O1 to O2 state and from the O2 to O1 state, respectively, of the phosphorylated ΔF508-CFTR-2RK. The number of events represents the sum of all opening and closing events. The relative frequency O1 to O2 and O2 to O1 transitions was expressed as percentage of the total number of openings.

Supplementary Table 4. CFTR gating energetics

O1	ΔF/2RK		ΔF/2RK+Hsc70/JA		ΔF/2RK		ΔF/2RK+Hsc70/JA		
	24 °C	SEM	24 °C	SEM	36 °C	SEM	36 °C	SEM	
$\Delta H^{\ddagger}_{O\text{-}C}$	37.5	17.2	16.9	4.2	37.5	17.2	16.9	4.2	kJ mol^{-1}
$\Delta S^{\ddagger}_{O\text{-}C}$	42.6	56.7	-28.9	14	42.6	56.7	-28.9	14	J mol^{-1}K^{-1}
$T\Delta S^{\ddagger}_{O\text{-}C}$	12.6	16.8	-8.6	4.1	13.2	17.5	-8.9	4.3	kJ mol^{-1}
$\Delta G^{\ddagger}_{O\text{-}C}$	24.9	16.9	25.5	8.4	24.4	16.7	25.8	8.5	kJ mol^{-1}
$\Delta H^{\ddagger}_{C\text{-}O}$	97.1	20.4	-4.4	16.5	-221.1	32.6	-4.4	16.5	kJ mol^{-1}
$\Delta S^{\ddagger}_{C\text{-}O}$	222.7	68.1	-116.7	54.5	-828.3	106.4	-116.7	54.5	J mol^{-1}K^{-1}
$T\Delta S^{\ddagger}_{C\text{-}O}$	66.2	20.2	-34.7	16.2	-256	32.9	-36.1	16.9	kJ mol^{-1}
$\Delta G^{\ddagger}_{C\text{-}O}$	30.9	7.7	30.2	25.3	35	4.8	31.6	26.1	kJ mol^{-1}
$\Delta H_{O\text{-}C}$	59.5	16.6	-21.3	20.8	-258.6	49.8	-21.3	20.8	kJ mol^{-1}
$\Delta S_{O\text{-}C}$	180.2	84.8	-87.7	41.3	-870.8	163.1	-87.7	41.3	J mol^{-1}K^{-1}
$T\Delta S_{O\text{-}C}$	53.5	25.2	-26.1	12.3	-269.2	50.4	-27.1	12.8	kJ mol^{-1}
$\Delta G_{O\text{-}C}$	6	2.7	4.8	2.9	10.6	3.8	5.8	3.5	kJ mol^{-1}

O2	ΔF/2RK		ΔF/2RK+Hsc70/JA		ΔF/2RK		ΔF/2RK+Hsc70/JA		
	24 °C	SEM	24 °C	SEM	36 °C	SEM	36 °C	SEM	
$\Delta H^{\ddagger}_{O\text{-}C}$	9.4	15.4	-7.7	24.2	9.4	15.4	-7.7	24.2	kJ mol^{-1}
$\Delta S^{\ddagger}_{O\text{-}C}$	-77.1	50.7	-132.1	80	-77.1	50.7	-132.1	80	J mol^{-1}K^{-1}
$T\Delta S^{\ddagger}_{O\text{-}C}$	-22.9	15.1	-39.3	23.8	-23.8	15.7	-40.8	24.7	kJ mol^{-1}
$\Delta G^{\ddagger}_{O\text{-}C}$	32.3	30.4	31.5	32.2	33.2	31	33.1	33.4	kJ mol^{-1}
$\Delta H^{\ddagger}_{C\text{-}O}$	97.1	20.4	-4.4	16.5	-221.1	32.6	-4.4	16.5	kJ mol^{-1}
$\Delta S^{\ddagger}_{C\text{-}O}$	222.7	68.1	-116.7	54.5	-828.3	106.4	-116.7	54.5	J mol^{-1}K^{-1}
$T\Delta S^{\ddagger}_{C\text{-}O}$	66.2	20.2	-34.7	16.2	-256	32.9	-36.1	16.9	kJ mol^{-1}
$\Delta G^{\ddagger}_{C\text{-}O}$	30.9	7.7	30.2	25.3	35	4.8	31.6	26.1	kJ mol^{-1}
$\Delta H_{O\text{-}C}$	87.7	29.5	3.3	11	-230.5	47.9	3.3	11	kJ mol^{-1}
$\Delta S_{O\text{-}C}$	299.8	118.8	15.5	8.4	-751.1	130.4	15.5	8.4	J mol^{-1}K^{-1}
$T\Delta S_{O\text{-}C}$	89.1	35.3	4.6	2.5	-232.2	40.3	4.8	2.6	kJ mol^{-1}
$\Delta G_{O\text{-}C}$	-1.4	0.9	-1.3	1.2	1.7	0.9	-1.5	1.4	kJ mol^{-1}

	WT		WT		
	24 °C	SEM	36 °C	SEM	
$\Delta H^{\ddagger}_{O\text{-}C}$	40	5.7	40	5.7	kJ mol^{-1}
$\Delta S^{\ddagger}_{O\text{-}C}$	34.9	18.9	34.9	18.9	J mol^{-1}K^{-1}
$T\Delta S^{\ddagger}_{O\text{-}C}$	10.4	5.6	10.8	5.9	kJ mol^{-1}
$\Delta G^{\ddagger}_{O\text{-}C}$	29.6	6.7	29.2	6.7	kJ mol^{-1}
$\Delta H^{\ddagger}_{C\text{-}O}$	47.8	7.5	47.8	7.5	kJ mol^{-1}
$\Delta S^{\ddagger}_{C\text{-}O}$	57.5	24.6	57.5	24.6	J mol^{-1}K^{-1}
$T\Delta S^{\ddagger}_{C\text{-}O}$	17.1	7.3	17.8	7.6	kJ mol^{-1}
$\Delta G^{\ddagger}_{C\text{-}O}$	30.7	7	30.1	6.9	kJ mol^{-1}
$\Delta H_{O\text{-}C}$	7.8	1.2	7.8	1.2	kJ mol^{-1}
$\Delta S_{O\text{-}C}$	22.6	10.6	22.6	10.6	J mol^{-1}K^{-1}
$T\Delta S_{O\text{-}C}$	6.7	3.2	6.7	3.2	kJ mol^{-1}
$\Delta G_{O\text{-}C}$	1.1	0.3	0.8	0.2	kJ mol^{-1}

The transitional and steady-state energetics of the phosphorylated WT- and ΔF508-CFTR-2RK channel gating were calculated as described in Methods. $\Delta H^{\ddagger}_{o\text{-}c}$, $\Delta S^{\ddagger}_{o\text{-}c}$, $T\Delta S^{\ddagger}_{o\text{-}c}$, and $\Delta G^{\ddagger}_{o\text{-}c}$ are transitional energetic parameters of closings. $\Delta H^{\ddagger}_{c\text{-}o}$, $\Delta S^{\ddagger}_{c\text{-}o}$, $T\Delta S^{\ddagger}_{c\text{-}o}$, and $\Delta G^{\ddagger}_{c\text{-}o}$ are transitional energetic parameters of openings. $\Delta H_{O\text{-}C}$, $\Delta S_{O\text{-}C}$, $T\Delta S_{O\text{-}C}$, and $\Delta G_{O\text{-}C}$ are hallmarks of the open state steady-state energetics relative to the closed state.

Supplementary Table 5. siRNA and antibodies

siRNA	Type	Cat#	Supplier	Target sequence	Ref
NT		D-001210-01	Dharmacon	UAGCGACUAAACACAUCAA	
HSP90AA1	siGENOME, SMARTpool	M-005186-02-0005	Dharmacon		
HSP90AB1	siGENOME, SMARTpool	M-005187-02-0005	Dharmacon		
HSP70	ON-TARGETplus, SMARTpool	L-005168-00-0005	Dharmacon		
DNAJA1	custom	custom	Dharmacon	GUGAAGGACUGUAAUCAUA	
DNAJA2	custom	custom	Dharmacon	CCACAAAGCUUUACAUCUU	
AHA1	custom	custom	Dharmacon	NNAUUGGUCCACGGAUAAGCU	[20]
HSC70		SI04236596	QIAGEN	TGGGCATTCTCAATACTTGAA	

Gene name	Ab	Dilution	Supplier
HA	Mouse mAb (901515), Anti-HA.11	1:2000	BioLegend
HA	Mouse mAb 660	1:2000	Gifted by Dr. Riordan
HA	Mouse mAb M3A7	1:500	Gifted by Dr. Riordan
Hsp90AA1 (HSP90a)	Rat mAb (9D2), ADI-SPA-840	1:2000	Enzo
Hso90AB1 (HSP90b)	Rabbit pAb (D6S182/FLJ26984), GTX101448	1:1000	GeneTex
Hsp70	Mouse mAb (C92F3A-5), ADI-SPA-810	1:1000	Enzo
DNAJA1	Mouse mAb (KA2A5.6), sc-59554	1:1000	SantaCruz
DNAJA1	Rabbit pAb (SPA-400)	1:200	Stressgene
DNAJA2	Rabbit pAb	1:500	Gifted by Dr. Young JC
Aha1	Mouse mAb (1A2-A8), H00010598-M01	1:1000	Abnova
Hsc70	Rat mAb(1B5), ADI-SPA-815	1:2000	Enzo
alpha-Tubulin	Mouse mAb (DM1A), T9026	1:10000	SIGMA
Na+/K+ ATPase	Mouse mAb (H-3), sc-48345	1:50000	Santacruz
Grp78/Bip	Rabbit pAb, PA1-014A	1:1000	Affinity Bioreagents
ErbB2	Mouse mAb, 9G6, ab16899	1:1000	Abcam

SUPPLEMENTARY REFERENCES

1. Vembar SS, Brodsky JL. One step at a time: endoplasmic reticulum-associated degradation. *Nature reviews Molecular cell biology* **9**, 944-957 (2008).

2. Wang W, Okeyo GO, Tao B, Hong JS, Kirk KL. Thermally unstable gating of the most common cystic fibrosis mutant channel (DeltaF508): "rescue" by suppressor mutations in nucleotide binding domain 1 and by constitutive mutations in the cytosolic loops. *J Biol Chem* **286**, 41937-41948 (2011).

3. Wang Y*, et al.* CFTR potentiators partially restore channel function to A561E-CFTR, a cystic fibrosis mutant with a similar mechanism of dysfunction as F508del-CFTR. *Br J Pharmacol*, (2014).

4. Veit G*, et al.* Some gating potentiators, including VX-770, diminish DeltaF508-CFTR functional expression. *Science translational medicine* **6**, 246ra297 (2014).

5. Okiyoneda T*, et al.* Peripheral protein quality control removes unfolded CFTR from the plasma membrane. *Science* **329**, 805-810 (2010).

6. Hegedus T, Aleksandrov A, Cui L, Gentzsch M, Chang XB, Riordan JR. F508del CFTR with two altered RXR motifs escapes from ER quality control but its channel activity is thermally sensitive. *Biochimica et biophysica acta* **1758**, 565-572 (2006).

7. Roxo-Rosa M*, et al.* Revertant mutants G550E and 4RK rescue cystic fibrosis mutants in the first nucleotide-binding domain of CFTR by different mechanisms. *Proc Natl Acad Sci U S A* **103**, 17891-17896 (2006).

8. Pissarra LS*, et al.* Solubilizing mutations used to crystallize one CFTR domain attenuate the trafficking and channel defects caused by the major cystic fibrosis mutation. *Chemistry & biology* **15**, 62-69 (2008).

9. Rabeh WM*, et al.* Correction of both NBD1 energetics and domain interface is required to restore DeltaF508 CFTR folding and function. *Cell* **148**, 150-163 (2012).

10. Thibodeau PH*, et al.* The cystic fibrosis-causing mutation deltaF508 affects multiple steps in cystic fibrosis transmembrane conductance regulator biogenesis. *J Biol Chem* **285**, 35825-35835 (2010).

11. Loo TW, Bartlett MC, Clarke DM. The V510D suppressor mutation stabilizes DeltaF508-CFTR at the cell surface. *Biochemistry* **49**, 6352-6357 (2010).

12. Amaral MD, Farinha CM. Rescuing mutant CFTR: a multi-task approach to a better outcome in treating cystic fibrosis. *Current pharmaceutical design* **19**, 3497-3508 (2013).

13. Liu X, O'Donnell N, Landstrom A, Skach WR, Dawson DC. Thermal instability of DeltaF508 cystic fibrosis transmembrane conductance regulator (CFTR) channel function: protection by single suppressor mutations and inhibiting channel activity. *Biochemistry* **51**, 5113-5124 (2012).

14. He L, Aleksandrov LA, Cui L, Jensen TJ, Nesbitt KL, Riordan JR. Restoration of domain folding and interdomain assembly by second-site suppressors of the DeltaF508 mutation in CFTR. *FASEB journal : official publication of the Federation of American Societies for Experimental Biology* **24**, 3103-3112 (2010).

15. Jih KY, Li M, Hwang TC, Bompadre SG. The most common cystic fibrosis-associated mutation destabilizes the dimeric state of the nucleotide-binding domains of CFTR. *The Journal of physiology* **589**, 2719-2731 (2011).

16. Cui L*, et al.* The role of cystic fibrosis transmembrane conductance regulator phenylalanine 508 side chain in ion channel gating. *The Journal of physiology* **572**, 347-358 (2006).

17. Csanady L, Mihalyi C, Szollosi A, Torocsik B, Vergani P. Conformational changes in the catalytically inactive nucleotide-binding site of CFTR. *J Gen Physiol* **142**, 61-73 (2013).

18. Rohl A, Rohrberg J, Buchner J. The chaperone Hsp90: changing partners for demanding clients. *Trends in biochemical sciences* **38**, 253-262 (2013).

19. Protasevich I*, et al.* Thermal unfolding studies show the disease causing F508del mutation in CFTR thermodynamically destabilizes nucleotide-binding domain 1. *Protein Sci* **19**, 1917-1931 (2010).

20. Wang X*, et al.* Hsp90 cochaperone Aha1 downregulation rescues misfolding of CFTR in cystic fibrosis. *Cell* **127**, 803-815 (2006).
